# Reconstructed historical distribution and phylogeography unravels non-steppic origin of *Caucasotachea vindobonensis* (Gastropoda: Helicidae)

**DOI:** 10.1007/s13127-017-0337-3

**Published:** 2017-09

**Authors:** Łukasz Kajtoch, Angus Davison, Adele Grindon, Tamás Deli, Gábor Sramkó, Mariusz Gwardjan, Sergei Kramarenko, Dominika Mierzwa-Szymkowiak, Rafał Ruta, Radosław Ścibior, János Pál Tóth, Chris Wade, Michał Kolasa, Roman V. Egorov, Zoltán Fehér

**Affiliations:** 1 Institute of Systematics and Evolution of Animals Polish Academy of Sciences, Sławkowska 17, 31-016 Krakow, Poland; 2 School of Life Sciences, University of Nottingham, University Park, Nottingham NG7 2RD, UK; 3 Directorate of Museums of Békés County, Munkácsy Mihály Museum, Széchenyi u. 9, Békéscsaba 5600, Hungary; 4 MTA-DE “Lendület” Evolutionary Phylogenomics Research Group, Debrecen, Hungary; 5 Department of Botany, University of Debrecen, Debrecen, Hungary; 6 The Wildlife Research and Conservation Society, Sienkiewicza 68, 25-501 Kielce, Poland; 7 Mykolayiv National Agrarian University, 9 Georgiy Gongadze Str, Mykolayiv 54020, Ukraine; 8 Museum and Institute of Zoology, Polish Academy of Sciences, Wilcza 64, 00-679 Warszawa, Poland; 9 Department of Biodiversity and Evoutionary Taxonomy, University of Wrocław, Przybyszewskiego 65, 51-179 Wrocław, Poland; 10 Department of Zoology, Animal Ecology and Wildlife Management, University of Life Sciences in Lublin, Akademicka, 13 20-950 Lublin, Poland; 11 MTA-DE “Lendület” Behavioural Ecology Research Group, Debrecen, Hungary; 12 Chkalova str., 5-32, Lobnya 141732, Russia; 13 Department of Zoology, Hungarian Natural History Museum, Baross utca 13, Budapest 1088, Hungary; 14 3rd Zoology Department, Natural History Museum Vienna, 1010 Burgring 7, Vienna, Austria

**Keywords:** Mollusca, Niche modeling, Demography, Pleistocene, Holocene, Steppe

## Abstract

Existing data on the phylogeography of European taxa of steppic provenance suggests that species were widely distributed during glacial periods but underwent range contraction and fragmentation during interglacials into “warm-stage refugia.” Among the steppe-related invertebrates that have been examined, the majority has been insects, but data on the phylogeography of snails is wholly missing. To begin to fill this gap, phylogeographic and niche modeling studies on the presumed steppic snail *Caucasotachea vindobonensis* were conducted. Surprisingly, reconstruction of ancestral areas suggests that extant *C. vindobonensis* probably originated in the Balkans and survived there during the Late Pleistocene glaciations, with a more recent colonization of the Carpatho-Pannonian and the Ponto-Caspian regions. In the Holocene, *C. vindobonensis* colonized between the Sudetes and the Carpathians to the north, where its recent and current distribution may have been facilitated by anthropogenic translocations. Together, these data suggest a possible non-steppic origin of *C. vindobonensis*. Further investigation may reveal the extent to which the steppic snail assemblages consist partly of Holocene newcomers.

## Introduction

Until recent anthropogenic habitat destruction, steppe and related xeric grasslands covered almost 10% of the land in Eurasia, stretching from the Pannonian Basin and Black Sea coast to Mongolia and Manchuria (McNeill 2011). The origin of steppe in particular is complex. “Cold steppes” were abundant during glacial periods in front of ice-sheets, whereas steppic patches were mixed with tundra elements in a so-called steppe-tundra environment (Nehring 1890; Willis and van Andel 2004), currently almost absent on Earth except north-east Asia and Alaska (Yurtsev 1982, 2000; Ehlers and Gibbard 2004). “Meadow steppes” were probably widespread during the Pleistocene in more southern and warmer areas in an extensive zone across Eurasia and currently are restricted to the steppe belt stretching from the Pannonian Basin and the Pontic region to Central Asia (Willis and van Andel 2004; Markova et al. 2009), with extrazonal analogs present locally in Central and Western Europe and the Balkans (called there xeric grasslands, calcareous grasslands, or xerothermic turfs) (Fekete et al. 2014; Pokorný et al. 2015). The origin of some dry grasslands and scrublands in continental Iberia, the Balkans, and Anatolia is probably independent from Eurasian steppes and more ancient (of Pliocene origin), although there is some evidence for connection even between Iberian and Asian steppes (Ribera and Blasco-Zumeta 1998; González-Sampériz et al. 2010).

All types of steppes and xeric grasslands sustain rich communities of plants and animals, with many species tightly related to these types of environments (e.g., Pärtel et al. 2005; Mazur and Kubisz 2013; Dengler et al. 2014). Many of these species, as well as the habitats and assemblages that they form (including dry lands, xeric grasslands, and steppic plant associations) are threatened and protected under the Natura 2000 network (http://ec.europa.eu/environment/nature/legislation/habitatsdirective/docs/2007_07_im.pdf).

Our knowledge of the phylogeography of European taxa of continental provenance (mainly steppic) is relatively low (Stewart et al. 2010; Varga 2010) compared with data collected for taxa from other types of environments in Europe. Recently, it has been summarized (Kajtoch et al. 2016) that steppic species show phylogeographic patterns which are generally discordant with that described for temperate species (Taberlet et al. 1998; Hewitt 2000, 2004; Schmitt 2007) but are instead similar to patterns found in cold-adapted artic-alpine species (Stewart and Lister 2001; Schmitt and Varga 2012). In contrast with temperate-adapted species, both cold- and xeric-adapted taxa seemed to have been widely distributed during glacial periods but underwent range contraction during interglacials, when their populations were restricted to refugial areas called “warm-stage” refugia (Schönswetter et al. 2005; Varga and Schmitt 2008; Holderegger and Thiel-Egenter 2008; Schmitt 2009; Stewart et al. 2010). Moreover, steppic taxa now show a high level of population genetic structure across their geographic ranges but generally lack variation at a local level (Kajtoch et al. 2016). This pattern suggests the long-term existence of their populations in situ, range fragmentation, and contraction into “warm-stage refugia” during interglacial periods with highly limited gene flow across larger distances and barriers (like mountains and forested areas) (e.g., some beetles—Kajtoch et al. 2009; rodents—Cserkész et al. 2016; Neumann et al. 2005; and plants—Durka et al. 2013; Cieślak 2014).

Among the steppe-related invertebrates that have been examined, the majority have been insects, especially butterflies and beetles (Bereczki et al. 2005; Wahlberg and Saccheri 2007; Rutkowski et al. 2009; Kajtoch et al. 2009; Kubisz et al. 2012; Przybyłowicz et al. 2014), and so little is known about other invertebrate groups. Gastropod mollusks in particular have been underrepresented in surveys despite the fact that speciose families such as the Vertiginidae, Pupillidae, Enidae, Bradybaeiidae, Hygromiidae, and Helicidae are important contributors to the diversity of xeric grassland communities (e.g., Schileyko and Rymzhanov 2013). It is uncertain if the phylogeographic patterns observed for the majority of steppic species are also characteristic for steppic snails.

To better understand the biogeographical history and distribution mechanisms of steppic landsnails, we selected a widespread and typical steppe-inhabiting species, *Caucasotachea vindobonensis* (C. Pfeiffer, 1828) (Neiber and Hausdorf 2015) (formerly known as *Cepaea vindobonensis*) as a study system. Based on the distribution, frequent in the Pannonian Basin and in the Pontic region but rarer towards the Balkans and the north-western edge of the distribution (see below, “Study system”), and the relationship to Caucasian congeners, we presumed that this species originates from the Ponto-Caspian part of the steppic zone and its present range is the result of an east to west expansion.

Our hypothesis was that if the glacial refugia for this species were also in the steppic zone (primarily in the Ponto-Caspian), then we expected the molecular genetic diversity to peak in the Ponto-Caspian zone and be lower in the supposedly peripheral Balkan and north-western areas. If correct, steppic gastropods (at least this species) could be another exception to the “southern refugia” principle (Taberlet et al. 1998; Hewitt 2000). Verification of these ideas would also allow for formal identification of putative refugial areas, as well as contribute to understanding how geographic/environmental barriers (i.e., the Carpathians) shaped expansion and current connectivity of populations, and the timing of events. To test our hypotheses, we used the combined methods of phylogeography, demography, and modeling past and current distribution of *C. vindobonensis*.

## Materials and methods

### Study system

**Fig. 1:**
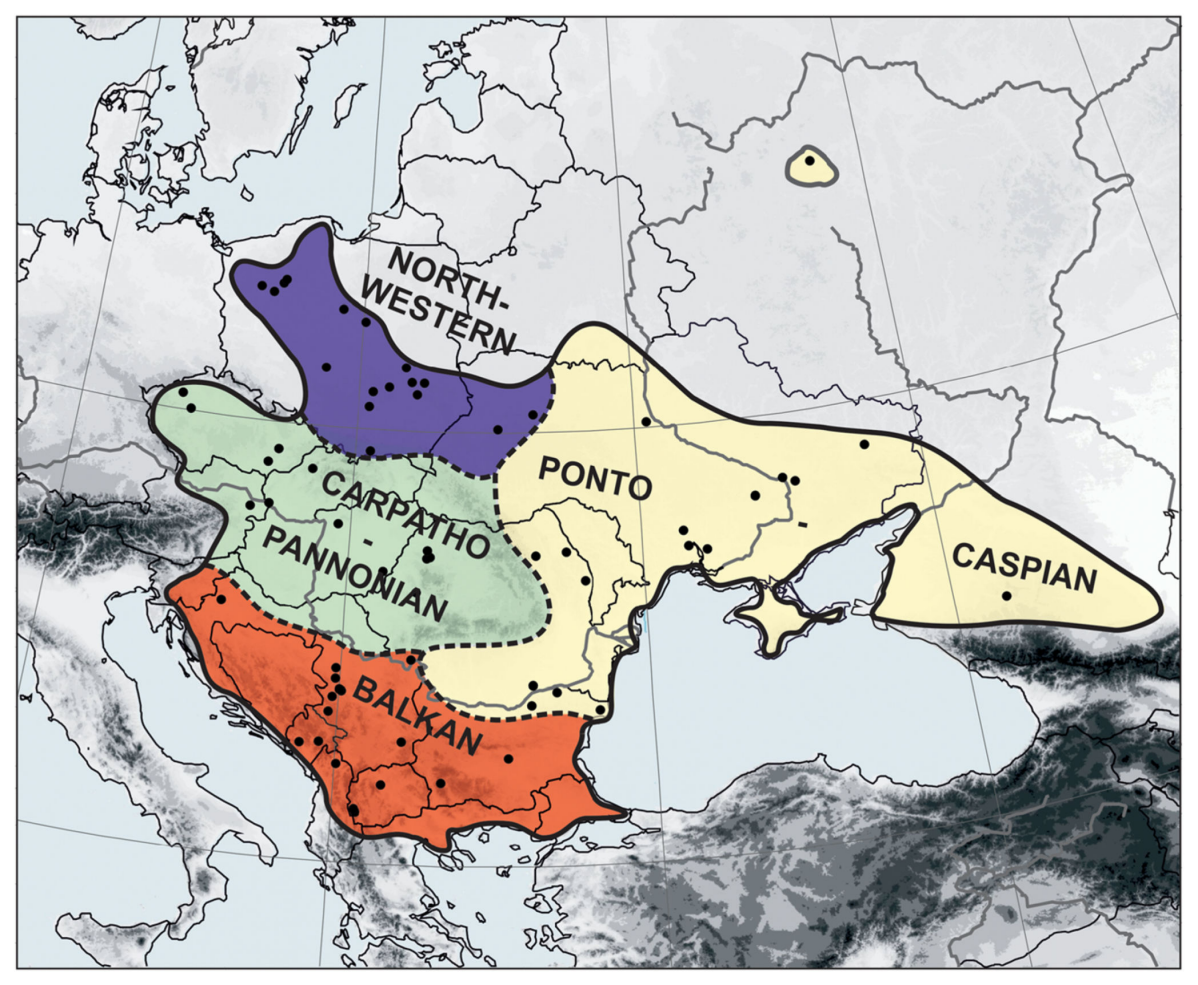
Range of the *Caucasotachea vindobonensis* with localization of sampling sites and defined regions of species distribution used for genetic analyses

*C. vindobonensis* is one of the most common and widely distributed land snails in the central, south-eastern and eastern parts of Europe (Fig. 1). Its current geographic range includes the two main European core steppe areas, the Ponto-Caspian steppe zone and the Pannonian Basin. To the west, its range extends to the eastern Alps and the Polish Lowland with some sporadic occurrences in Germany. To the east, it reaches the north-eastern foothills of the Caucasus Mts. To the south, it reaches the Balkan Peninsula except the southernmost parts (Soós 1943; Ložek 1964; Klemm 1973; Kerney et al. 1983; Welter-Schultes 2012; Kurtaev et al. 2012). Far in the north-east, there are some isolated occurrences in the Moscow region due to obvious human introductions (Egorov 2014; Schikov 2016).

According to our current understanding, *C. vindobonensis* is a typical steppic and forest-steppic species (Ložek 1964), which can inhabit also various types of xeric grasslands, arid and humid scrublands, forest edges, tallgrass vegetation and gallery forests along stream and river sides, as well as ruderal habitats (Soós 1943; Welter-Schultes 2012). Based on the distribution and local frequency, the snail is believed to be a predominantly central-eastern European species (Ložek 1964). Although it was long classified within the genus *Cepaea* Held, 1838 (Kerney et al. 1983), recent molecular evidence has shown that the species should better be placed in the genus *Caucasotachea* (Neiber and Hausdorf 2015; Neiber et al. 2016). As all other *Caucasotachea* species are distributed around the Caucasus and Mt. Elbourz (Neubert and Bank 2006; Welter-Schultes 2012), this implies a Caucasian origin of the genus (Neiber and Hausdorf 2015; Neiber et al. 2016).

### Sampling

*C. vindobonensis* specimens were collected during numerous field trips between 2005 and 2015. Moreover, some older samples were loaned from collections. In total, 139 snails from 56 localities were collected to represent the whole distribution range (Supplementary Table 1 and Supplementary File Fig. 1). Most specimens were preserved in 99% ethanol and frozen upon arrival at the laboratory, although some, not collected for molecular studies, were stored in 70–80% ethanol.

### Laboratory procedures

Small fragments of snail foot tissue were used for DNA extraction using a Nucleospin Tissue kit (Macherey-Nagel). Amplification and sequencing of fragments of a mitochondrial cytochrome oxidase I (COI) gene fragment was performed using either the primers of Folmer et al. (1994) or the alternative primers of Gittenberger et al. (2004). Additionally, six COI sequences belonging to this species, as well as to three selected outgroup species: *Macularia sylvatica*, *Causacotachea atrolabiata*, and *Causacotachea intercedens*, were downloaded from GenBank (accession numbers for outgroup taxa, respectively, KR705039, KT794388, and KR705044). The concentration of the reagents used for the amplification of all markers and the cycling profile for PCR were as in Grindon and Davison (2013). After purification using NucleoSpin Extract II (Macherey-Nagel), the PCR fragments were sequenced using a BigDye Terminator v.3.1. Cycle Sequencing Kit (Applied Biosystems) and run on an ABI 3100 Automated Capillary DNA Sequencer. All newly obtained sequences were deposited in GenBank (accession numbers are provided in Supplementary Table 1).

### Population genetics and demography

COI sequences were collected from 145 snails (one to six specimens, usually two to three, from each locality). Sequences were checked and aligned using BioEdit v.7.0.5.2 (Hall 1999) and ClustalX (Thompson et al. 1997). The final alignment had 620 characters and no gaps. Haplotypes were identified and standard genetic indices, such as number of variable sites (V), number of segregating sites (S), haplotype diversity (Hdiv), nucleotide diversity (π), and number of private haplotypes (Hpriv) for populations, were computed with DnaSP v.5 (Librado and Rozas 2009).

For some analyses, sequences were grouped according to their geographic provenance based on previously deducted (Kajtoch et al. 2016), distinct phylogeographic units of steppic organisms: (i) Balkan Peninsula (samples from Dinaric Mts. in Croatia, Serbia, Montenegro, Macedonia and Albania, and the Balkan and Pirin Mts. in Bulgaria), (ii) Carpatho-Pannonian (Romania-Transylvania, Hungary, Austria, Czech Republic, Slovakia), (iii) Ponto-Caspian (Central and Eastern Ukraine, Southern Russia, Dobrogea in Romania and Bulgaria), and (iv) north-western (Poland and Western Ukraine-Podolia) (Fig. 1).

To test if examined populations experienced expansion and/or contraction events in their history, the mismatch distribution (MD) (Rogers and Harpending 1992) was calculated in Arlequin v.3.5 (Excoffier and Lischer 2010) for the pre-defined regional groups of populations.

### Phylogenetics

A phylogenetic tree was reconstructed for the haplotype data, using a maximum likelihood (ML) approach implemented in PhyML 3.0 software (Guindon et al. 2010) and the online interface at http://www.atgc-montpellier.fr/phyml/ (Supplementary Fig. 1). The beta version of the program was used, which includes automatic selection of the best model of DNA substitution called Smart Model Selection (http://www.atgc-montpellier.fr/sms/) using Akaike information criterion (AIC). Branch support was obtained by the approximate likelihood-ratio test (aLRT) (Anisimova and Gascuel 2006), which is a likelihood-based alternative to computationally intensive bootstrapping. A molecular clock test was performed by comparing the ML value for the given topology, with and without the molecular clock constraints under the GTR model in MEGA6 (Tamura et al. 2013). The null hypothesis of equal evolutionary rate throughout the tree was rejected at a 5% significance level. As an ultrametric tree was required for the subsequent analyses, the ML tree was transformed into an ultrametric one under the relaxed clock model (lambda was set to “0”) using the chronos() function of the APE package (Paradis et al. 2004) of the R software (R Development Core Team 2014).

Haplotype networks were constructed using the median-joining algorithm (MJ) (Bandelt et al. 1999) in the software PopArt (http://popart.otago.ac.nz/) for the haplogroups defined from the topology of ML tree (as above).

### Identification of ancestral areas

The reconstruction of past geographic ranges was performed with Lagrange (Ree and Smith 2008), which uses a dispersal-extinction-cladogenesis (DEC) modeling for analyzing ML probabilities of rate transitions as a function of time. Geographical subdivision of samples followed the same as for the intraspecific variability analyses (Fig. 1; Supplementary Table 1). The input file was prepared in Lagrange Configurator (http://www.reelab.net/lagrange/configurator) using the ultrametric tree described above, and the haplotype-distribution matrix (Supplementary Table 2). Distribution and extinction rates were estimated. Range constraint was set either to “2” or “3” (i.e., ancestral lineages were allowed to occupy no more than 2 or 3 geographical subunits at the same time), and migration was permitted either with the same dispersal probabilities (“1. 0”) between any of the four subunits, or lower probabilities (“0.5” and “0.25”) were assigned to migration between the non-adjacent areas (BK and NW). We tested six combinations of the two range and three dispersal constraints to see which one had the highest likelihood.

### Distribution modeling

MaxEnt modeling approach was used to predict the potential distribution of *C. vindobonensis* using BIOCLIM variables (Busby 1991). MaxEnt is a widely used method for predicting species distributions using presence-only data (Phillips et al. 2006; Warren and Seifert 2010).

In addition to the recently collected material, further distribution records were taken from the following public collections: Hungarian Natural History Museum Budapest (HNHM), Natural History Museum Vienna (NHMW), Munkácsy Mihály Museum, Békéscsaba (MMM), The Zoological Museum of the Zoological Institute of Russian Academy of Sciences, St. Petersburg (ZIN), Zoological Museum of Moscow State University (ZMMU), and Museum and Institute of Zoology Polish Academy of Sciences (MIZ PAS) and from the private collections of Frank Walther (Hamburg, Germany) and Sergei Kramarenko (Mykolayiv, Ukraine). See the detailed list of overall 393 distribution records in Supplementary Table 3.

To counterbalance sampling bias in the presence data, the package “spThin” was used for systematic sampling (Aiello-Lammens et al. 2015) in an R computing environment (R Development Core Team 2014). This method makes a subsample of records with geographically even distribution. Systematic sampling proved to be consistently ranked among the best-performing method in a recent comprehensive study by Fourcade et al. (2014). Finally, 100 non-overlapping presence points were used for modeling the distribution (typed in bold in Supplementary Table 3). Climate variables were downloaded from the WorldClim database (www.worldclim.org). Although MaxEnt is more robust in controlling for correlations between variables than stepwise regression (Elith et al. 2011), strongly correlated variables (*r* > 0.75) are recommended to be excluded from the analysis (see: Elith et al. 2010; Stohlgren et al. 2010). ENMtools 1.4 was used to calculate the level of correlations (Warren et al. 2010). To assess which predictors provide the most useful information by itself, we applied jackknife test using MaxEnt. The results of the jackknife test, the correlation tests, and the biological knowledge on *C. vindobonensis* were considered during variable selection. Finally, Akaike information criterion in ENMtools 1.4 was used to select the best model from the alternatives.

The discrimination ability of the model was evaluated by area under the curve (AUC) measure. The value of AUC varies between 0.0 and 1.0, where 1.0 is considered to be a perfect prediction, while a value ≤0.5 is considered not being better than a random prediction (Fielding and Bell 1997; Franklin and Miller 2009). The distribution models were projected back to the Last Glacial Maximum (LGM, i.e., *ca* 21,000 years before present). For the projections, we used the predictions of two different global circulation models (MIROC-ESM and CCSM4).

## Results

### Population genetics and demography

Standard genetic indices for *C. vindobonensis* are summarized in Table 1. All examined groups of regional populations expressed similar levels of genetic diversity, except for the geographically distant, introduced population from Moscow, which was characterized by a single haplotype, closely related to haplotypes from Ukraine.

The distribution of pairwise differences (Supplementary Fig. 2) was multimodal for Carpatho-Pannonian and Ponto-Caspian MD, but unimodal for the Balkan and north-western regions. Moreover, a left-shifted histogram for north-western populations could indicate recent expansion in that region, and a right-shifted histogram for Balkan populations could suggest the past expansion in these populations. Multimodal histograms generated for Carpatho-Pannonian and Ponto-Caspian populations could suggest multiple expansion events in these regions, in different periods and from different refugia. However, a low and statistically non-significant Harpending’s raggedness index and a low SSD value (Supplementary Table 4) show that demographic and spatial expansions could not be rejected.

### Phylogeography

**Fig. 2:**
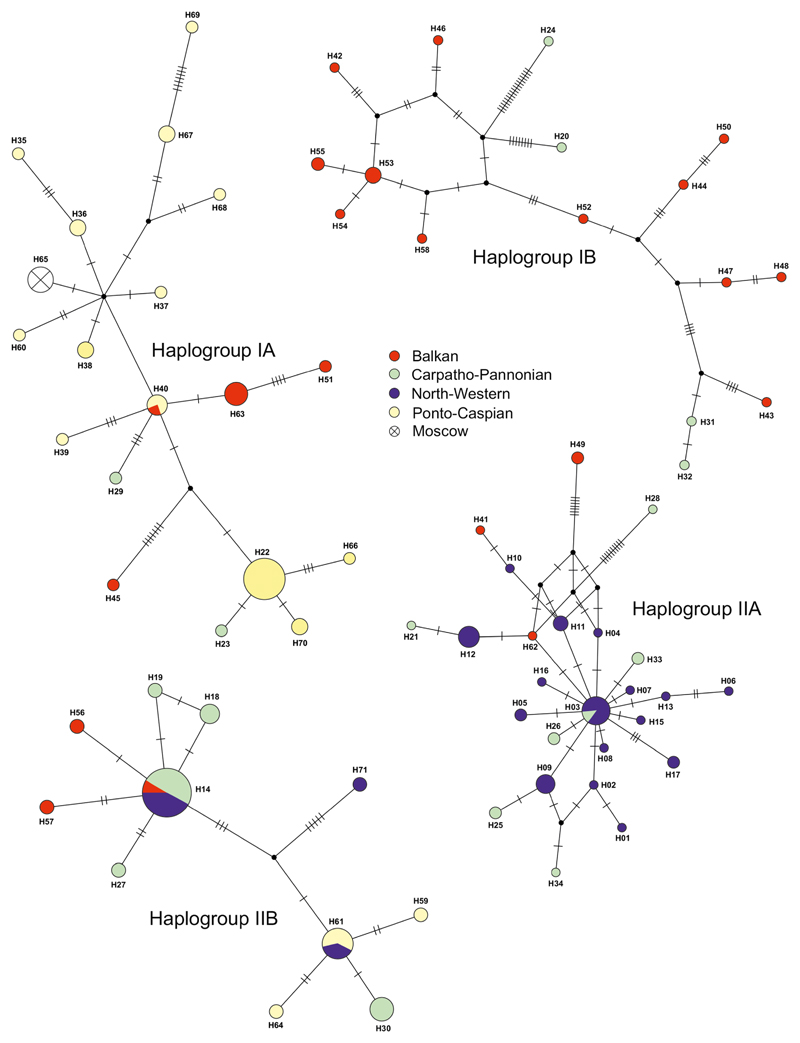
Median-joining networks of *Caucasotachea vindobonensis* COI haplotypes with assignment to the defined regions of species distribution

**Fig. 3:**
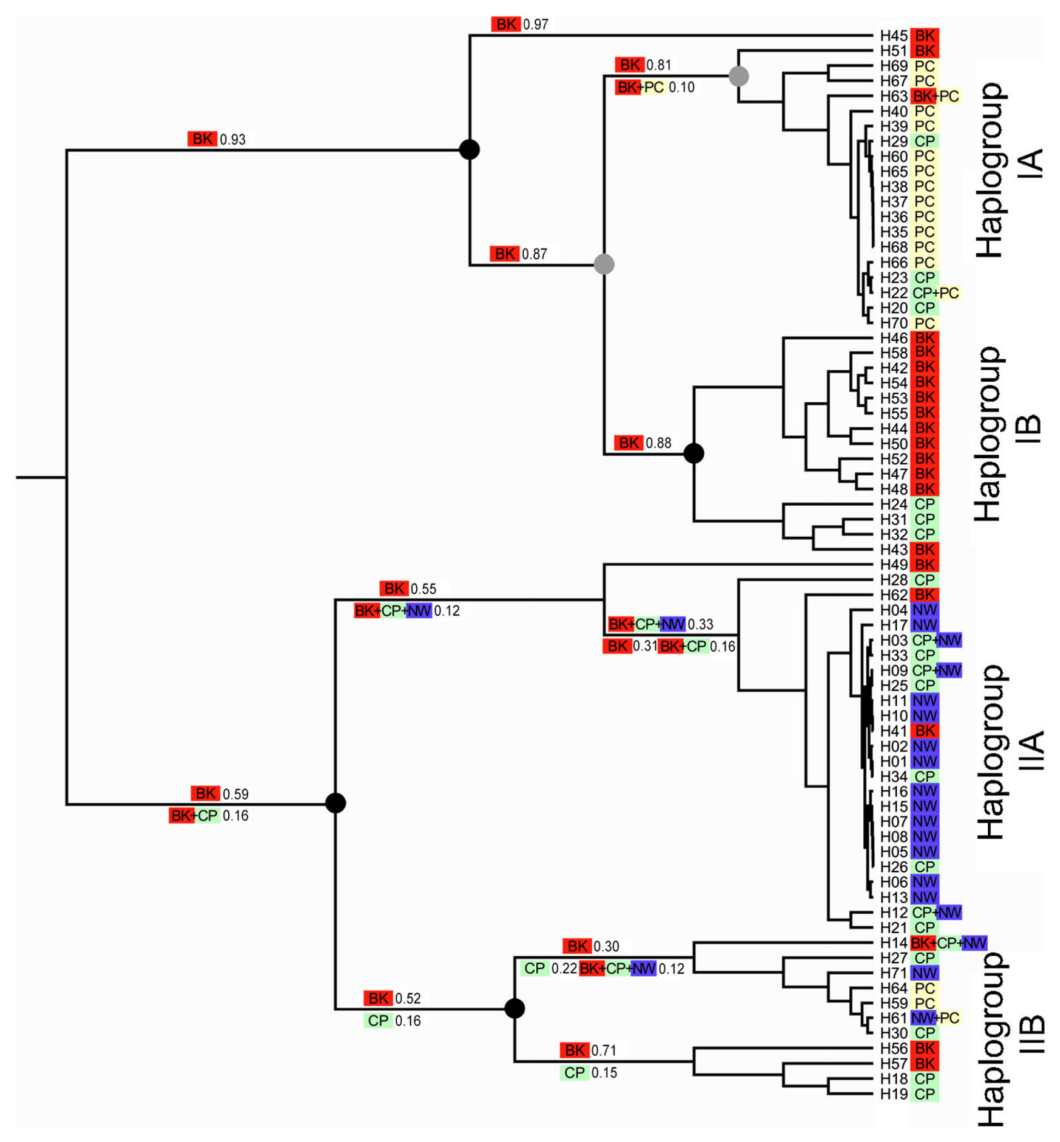
Reconstruction of the geographic range evolution. For the geographic position of the samples and the subdivision of the range, see Fig. 1. The ultrametric ML tree shows phylogenetic relationships of the *Caucasotachea vindobonensis* haplotypes based on COI sequences. High branch supports for the main clades are indicated by *black/gray dots* (*gray* aLRT >0.80; *black* aLRT >0.95). The *colored symbols at the tips* indicate the current geographic origin of the haplotypes (see also Supplementary Table 1). Values at the branches indicate all alternative scenarios with likelihoods above 10% for the origin of the clades’ common ancestors (*BK* Balkans, *CP* Carpatho-Pannon, *PC* north-western, *NW* Ponto-Caspian). The ancestors were allowed to occupy a maximum of three geographic areas. Migration was permitted between all regions, but lower probability (“0.25” instead of “1.0”) was assigned in the dispersal constraints for migration between areas not being immediately adjacent (between north-western and the Balkans)

The ML tree suggests the presence of two main lineages, each of which consist of two haplogroups, respectively (Supplementary Fig. 1; Figs. 2 and 3). Haplogroup IA (hgIA) contains haplotypes found mainly in the Balkans. Haplogroup IB (hgIB) contains haplotypes found mainly from Ponto-Caspian region with some haplotypes from the Carpatho-Pannonian and the Balkan regions. Haplogroup IIA (hgIIA) contains haplotypes found mainly in the north-western populations with single haplotypes from both the Carpatho-Pannonian and the Balkan region. Haplogroup IIB (hgIIB) contains haplotypes found in all regions, but most frequently from the Carpatho-Pannonian area.

To identify the ancestral region, six combinations of the two range and three dispersal constraints were compared. The model with a range constraint of maximum three sub-areas and lower migration rate (0.25) between non-adjacent areas (i.e., between north-western and the Balkans) had the highest likelihood (Fig. 3); however, all settings produced largely similar results. For most clades, including the two main ones at the basal split, the Balkan origin seems to be the most likely, or sometimes the only likely scenario (Fig. 3). The only exception is the clade that contains the majority of north-western haplotypes (haplogroup IIA of the haplotype network). For this group, the Balkan origin is only the second most likely scenario, following the scenario that the clade’s ancestor occupied three sub-areas, namely the Balkans, the Carpatho-Pannon and the north-western region.

### Distribution modeling

**Fig. 4:**
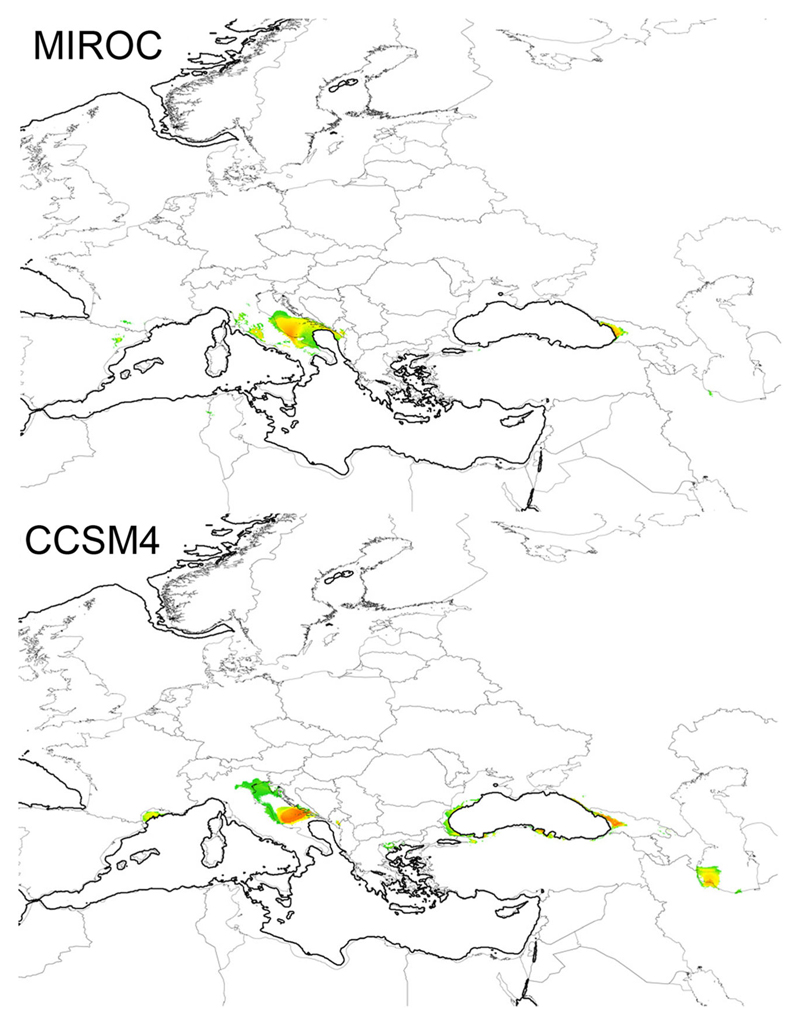
Potential distribution of *Caucasotachea vindobonensis* during the LGM based on different climate models (CCSM4, MIROC-ESM). Warmer/darker colors indicate more suitable climatic conditions

Based on AIC values, a model with four variables was selected: temperature annual range (bio7), mean temperature of warmest quarter (bio10), precipitation seasonality (bio15), and precipitation of warmest quarter (bio18). The models received excellent support values (mean AUC = 0.967, standard deviation = 0.021) following the nomenclature of Swets (1988) (Supplementary Table 5). The predicted present distribution of *C. vindobonensis* yielded a good fit for the known area, although it also predicts suitable areas to the Apennine Peninsula, the Iberian Peninsula, and northern Anatolia, where the species does not occur (Supplementary Fig. 3). Potential refugia during the LGM were predicted to occur in Southern France, the Adriatic Sea, the Balkans, the coastline of the Black Sea, and the Elburz Mountains (Fig. 4). Interestingly, most of these potential refugia are situated in areas currently under water but were dry lands during the LGM. The prediction of the climate models used (MIROC-ESM, CCSM4) showed similar predictions, although MIROC-ESM predicted smaller areas and more southerly positions than CCSM4 in most cases.

## Discussion

### History of origin of *C. vindobonensis*

*C. vindobonensis* was formerly classified in the genus *Cepaea*, and therefore, it was considered the easternmost member of that western European genus (Soós 1943; Kerney et al. 1983). This point of view has now completely changed because molecular phylogenetic evidence has rejected the monophyly of former *Cepaea* (Neiber and Hausdorf 2015), instead placing *C. vindobonensis* into *Caucasotachea.* As all other members of *Caucasotachea* are distributed in the Caucasian mountains and along the coasts of the eastern Black Sea and western Caspian Sea (Neiber and Hausdorf 2015; Neiber et al. 2016), it is likely that the group originated in that region. Oldest (Middle Miocene) fossil records conchologically resembling modern *C. vindobonensis* are from Crimea (Egorov, unpublished data), which provide further support for this assumption. Based on scattered fossils, it seems that at the end of the Pliocene, the range of this species already included Daghestan (Caucasus Mts) (Kurtaev et al. 2012). All other fossil records of this species are known from the uppermost Pliocene of Romania and Hungary (Soós 1943), Bulgaria (Alexandrowicz 2009), and southern Ukraine (Kunica 2007).

We can speculate that this species went through numerous range expansions and contractions during the Pleistocene, in concordance with glaciations and warmer periods. COI data implies the presence of at least two refugia during the last glaciation, both of which could have been in the Balkans. Demographic analyses suggest that *C. vindobonensis* first expanded in the Balkans, followed by the expansion into Carpatho-Pannonian and Ponto-Caspian regions. The most recent expansion, probably during the Holocene, was the expansion to the north of the Carpathians.

According to our reconstruction of historical areas, there were potential refugial areas during the LGM: (i) along the western coast of the Black Sea (Thrace); (ii) in western Transcaucasia (Colchis); and (iii) in the Adriatic Basin (now covered by sea but above the sea level during the LGM; Bortolami et al. 1977). Among these potential refugial areas, the first (Thrace) seemed to be the most probable. If *C. vindobonensis* had survived in an Adriatic refugium, it might have next expanded to the Po Valley and Apennine Peninsula—but it did not. Colchis as a refugium is questionable, as there are no past or current signs of *C. vindobonensis* being present there; however, it is within the area of origin for *Caucasotachea* snails. Predicted Last Glacial Maximum range did not include the Pannonian Plain, the Podolian Upland, the Ponto-Caspian Plain, and the areas north of them. Based on this, we suppose that the colonization of the Carpatho-Pannonian and the Ponto-Caspian regions started not earlier than the late Vistulian (also known as Würm) glacial. The difference in the two regions’ genetic heterogenity is due to the fact that the former one could have been colonized from both Balkan refugia, whereas the latter one only from the Eastern Balkan refugium. This multiple colonization of the Carpatho-Pannonian area, and presence of haplotypes from diverged lineages there, could bias expansion time estimates in that area.

The relatively lower genetic diversity of Pontic populations of *C. vindobonensis*, together with the low support for presence of ancestral area for this species in that region, all suggest that the Pontic region was probably not a refugium. Instead, the Pontic region was likely settled from eastern Balkan populations. These findings are generally congruent with the recent population genetic study of Snegin and Snegina (2016) on *C. vindobonensis*. Based on nuclear loci (allozymes, Random Amplification of Polymorphic DNA, Inter-Simple Sequence Repeats), they showed that Pontic populations of this species had reduced allelic diversity and increased levels of inbreeding in some groups and high levels of isolation of populations compared with Pannonian (Austrian) populations. This genetic pattern is in contrast with the expectations of an east-to-west expansion (in other words, eastern refugia) and concordant with a recent review of steppic species phylogeography in Eastern Central Europe (Kajtoch et al. 2016). Reverse order of origin and expansion (from the Pannonian/Balkan region to the Pontic region) has been observed for some steppic plants (e.g., *Pulsatilla patens*, Szczecińska et al. 2016) and rodents (e.g., *Sicista subtilis* agg., Cserkész et al. 2016).

Molecular and subfossil records are concordant for populations north of the Carpathians. The shell of this species are only known from Late Holocene deposits in southern Poland (Alexandrowicz 2013), consistent with our estimations of its recent expansion there. Interestingly, paleontological evidence suggests a larger distribution in northern areas during the last interglacial as it reached to Thüringien in central Germany (Soós 1943).

Our finding that the most probable refugia of *C. vindobonensis* were in the Balkans, and considering also its wide habitat preferences to contrasting types of xeric (grasslands, scrublands, open woods) and mesic environments (e.g., gallery forests), could also be interpreted as the strong argument against its strict steppic affinity. *C. vindobonensis* is perhaps better considered a species of southeast European (Balkan) origin, associated with rather more humid habitats characteristic for river valleys and gorges. It has probably expanded into more xeric environments of similar climatic conditions and plant communities in central and eastern Europe, including steppes. This pattern is supported by the fact that the species is currently more abundant in Pontic and Pannonian regions than in the Balkans and is still expanding to the north, e.g., in Poland (Mierzwa-Szymkowiak 2012). Examples of Mediterranean-like species in steppic fauna and flora of Eastern Central Europe have also been highlighted recently (Kajtoch et al. 2016). Steppic communities do not always have only Ponto-Caspian or Pannonian origin (see above), but apparently can have eastern-Mediterranean provenience, like the species complex of *Lacerta viridis* (Joger et al. 2007; Böhme et al. 2007) or the species complex of *Nannospalax leucodon* (Hadid et al. 2012; Kryštufek et al. 2012). However, these species have ranges restricted mainly to the Balkans, Pannonian Basin, and Black Sea coast only, usually not reaching areas north to the Carpathians and Eastern Europe.

Ideally, we would have also generated data from nuclear markers to support our analysis of mitochondrial DNA. Unfortunately, we found insufficient variation in ribosomal internal transcribed spacer (ITS) markers or a histone intron that has been used fairly widely in the phylogenetics of land snails (e.g., Cadahía et al. 2014). We also failed to cross-amplify DNA fragments from the restriction site associated DNA markers library generated on *Cepaea nemoralis* genomic DNA (Richards et al. 2013) and so are unable to corroborate the findings using other genetic markers, as is desirable (Toews and Brelsford 2012). Thus, the phylogeographic conclusions provided above are based on a single mitochondrial DNA fragment, and so should be treated with caution. Indeed, drawbacks (e.g., mito-nuclear discordance) of using a single nuclear marker have been highlighted in numerous studies and reviews (e.g., Hurst and Jiggins 2005; Galtier et al. 2009; Toews and Brelsford 2012). Relating specifically to land snails, the majority of phylogeographic studies have been based on single loci, almost exclusively mtDNA (e.g., Pfenninger and Posada 2002; Grindon and Davison 2013), and relatively rarely have other nuclear markers been used. In the few available studies which used both mitochondrial and nuclear DNA sequences, the patterns have been concordant (e.g., Pfenninger et al. 2003; Jesse et al. 2011; Harl et al. 2014). In our case, although we only used one marker, the conclusions are additionally supported by the use of niche modeling approach, which has become a standard complementary method alongside molecular studies (Scoble and Lowe 2010; Alvarado-Serrano and Knowles 2014).

### Current distinctiveness of populations

The geographic distribution of haplotypes showed that *C. vindobonensis* populations are structured spatially, but there are also signs of interregional gene flow. Mitochondrial haplogroups generally link snails from distinct geographic localities clustered in defined geographic regions (i.e., haplotypes from the same clade are coming from the same geographic region but from distinct localities). Moreover, in several cases, the same haplotypes were found in snails from highly distant localities belonging to different regions, especially in both the Carpatho-Pannonian and north-western populations. This is consistent with one of major routes of expansion/migration of steppic fauna and flora via the Moravian Gate, localized between the Carpathians and the Sudetes (e.g., Liana 1987; Mazur 2001). Another important route of expansion of steppic elements into Poland is from the east—from the Pontic region across Podolian Upland (Liana 1987; Mazur 2001). Apparently, this eastern route was insignificant in the case of *C. vindobonensis* as even the westernmost Ukrainian populations (from Podolian Upland) must have been settled from the reverse direction—from the Pannonian lineage via Poland but surprisingly not from adjacent populations in Pontic region. There are also other examples of rare haplotype sharing between geographically distant populations from Carpatho-Pannonian and Balkan, Balkan and Ponto-Caspian, Carpatho-Pannonian and Ponto-Caspian, and north-western and Ponto-Caspian regions. These shared haplotypes could be just examples of ancestral polymorphism present in various populations within the species or else they could also be explained in the light of dispersal of individuals.

### Human-assisted movement of snails?

It should also be considered that the current distribution of *C. vindobonensis* in some locations (especially in sites far from main range) is likely due to human translocations. A role of humans in the current distribution of other snails has been suggested previously (e.g., Lubell 2004; Cook and Peake 1960; Grindon and Davison 2013). Similarly, Kramarenko (2014) showed that terrestrial mollusks (including *C. vindobonensis*) can disperse passively by humans (anthropochory) even over large distances. Certainly, anthropochory is the most likely explanation for populations in the Moscow vicinity, ~550 km from the nearest natural populations of this snail in southern Russia (Egorov 2014; Schikov 2016). Only a single haplotype was found in that population, which is closely related to haplotypes from the Ponto-Caspian region. Similarly, the establishment of some populations in northern Poland and Germany could be of anthropogenic origin (Mierzwa 2009). The presence of several haplotypes found simultaneously in some populations from Carpatho-Pannonian and north-western regions, which are distant by 200–500 km and isolated by barriers (e.g., higher parts of the Carpathians), strongly suggests that expansion of this snail to the north could either be facilitated indirectly by human activity (e.g., habitat degradation at the landscape level) or directly caused by transfer of snails unintentionally (i.e., this could be connected with presence of Paleolithic trade routes called the “Amber route” (Bukowski 1988; Kwiatkowska and Manasterski 2013). These routes run from southern Europe via the Pannonian Basin to the Baltic coast, along which different products were carried, including some plants (e.g., grapes), serving as a means for snail transport. Some rare cases of shared haplotypes between remote regions (e.g., Balkan and Ponto-Caspian) could also be connected to either natural long-distance dispersion or human activity which was shown to be effective in transfer of land snails to overseas on ships in antiquity (Welter-Schultes 2008).

### Summary

We examined the mitochondrial phylogeography of *C. vindobonensis*, complemented with niche distribution modeling.

– Despite the Caucasian congeners suggesting an eastern (Pontic) origin for *C. vindobonensis*, we have found it more probable that the glacial refugial areas of this species were situated in the Balkans According to the most likely scenario, it colonized the Pannonian Basin and the Ponto-Caspian region during Late Pleistocene, reaching the Polish Lowland at the beginning of Holocene.
– *C. vindobonensis* does not follow the pattern previously identified in other strict steppic taxa, which were rather more common across Eastern Central Europe during glacial periods than interglacials. However, as *C. vindobonensis* was not found to be a steppic species in a strict sense (i.e., not just having a center of distribution in the steppic region, but also having a refugium there), further studies of other steppic mollusks, especially those with ranges restricted to steppic region (e.g., *Helicopsis striata* or *Chondrula tridens*) are necessary to investigate if this is a general pattern. For the moment, we hypothesize that steppic snail assemblages are formed by species of heterogenous origin: many Holocene newcomers, like *C. vindobonensis* and species are of actual steppic provenance.
– The genetic distinctiveness of *C. vindobonensis* populations from different regions in central, eastern, and south-eastern Europe is rather low and unfavorable areas like high mountain ranges do not constitute effective barrier for gene flow among distant localities. This is also discordant with general patterns found for steppic taxa.
– Expansion of this species during prehistoric and current times could have been facilitated by unintentional man-made transfers of snails across large distances.

## Supplementary Material

**Electronic supplementary material** The online version of this article (doi:10.1007/s13127-017-0337-3) contains supplementary material, which is available to authorized users.

Supplementary figure 1 Maximum likelihood phylogenetic tree reconstructed on haplotypes found in *Caucasotachea vindobonensis* populations. Values indicate branch supports

Supplementary figure 2 Mismatch distribution calculated for four regional groups of *Caucasotachea vindobonensis* populations

Supplementary figure 3 The potential distribution of *Caucasotachea vindobonensis*. *Warmer colors* indicate more suitable climatic conditions, while *crosses* indicate the subsample of presence points used for modeling (for more detail see material and methods)

Supplementary file 1

Supplementary table 1

Supplementary table 2

Supplementary table 3

Supplementary table 4

Supplementary table 5

## Figures and Tables

**Table 1:** Standard genetic indices of COI gene calculated for studied *Caucasotachea vindobonensis* regional groups of populations

Populations	*N*	*V*	*S*	*H*num	*H*priv	*H*priv/*H*num (%)	*H*div (SD)	πdiv (SD)	*D*
All	145	99	60	71	–	–	0.973 (0.006)	0.0205 (0.0071)	0.021
Balkan	29	50	34	23	20	87.0	0.980 (0.015)	0.0194 (0.0069)	0.020
Carpatho-Pannonian	37	63	38	21	16	76.2	0.920 (0.035)	0.0197 (0.0077)	0.020
North-western	41	31	22	19	14	73.7	0.917 (0.028)	0.0074 (0.0039)	0.008
Ponto-Caspian	38	37	25	18	14	77.8	0.910 (0.033)	0.0111(0.0047)	0.011

*N* sample number, *V* number of variable sites, *S* number of segregating sites, *H*num haplotype number, *H*priv number of private haplotypes, *H*div haplotype diversity, πdiv nucleotide diversity, *SD* standard deviation, *D* mean pairwise differences

## References

[R1] Aiello-Lammens ME, Boria RA, Radosavljevic A, Vilela B, Anderson RP (2015). spThin: an R package for spatial thinning of species occurrence records for use in ecological niche models. Ecography.

[R2] Alexandrowicz SW (2009). Malacofauna of Late Quaternary deposits from Muselievo, Northern Bulgaria. Folia Malacologica.

[R3] Alexandrowicz WP (2013). Late Glacial and Holocene molluscan assemblages in deposits filling palaeolakes in northern Poland. Studia Quaternaria.

[R4] Alvarado-Serrano DF, Knowles LL (2014). Ecological niche models in phylogeographic studies: applications, advances and precautions. Molecular Ecology.

[R5] Anisimova M, Gascuel O (2006). Approximate likelihood-ratio test for branches: a fast, accurate, and powerful alternative. Systematic Biology.

[R6] Bandelt HJ, Forster P, Röhl A (1999). Median-joining networks for inferring intraspecific phylogenies. Molecular Biology and Evolution.

[R7] Bereczki J, Pecsenye K, Peregovits L, Varga Z (2005). Pattern of genetic differentiation in the *Maculinea alcon* species group (Lepidoptera, Lycaenidae) in Central Europe. Journal of Zoological Systematics and Evolutionary Research.

[R8] Böhme MU, Fritz U, Kotenko T, Džukić G, Ljubisavljević K, Tzankov N, Berendonk TU (2007). Phylogeography and cryptic variation within the Lacerta Viridis Complex. Zoologica Scripta.

[R9] Bortolami GC, Fontes JC, Markgraf V, Saliege JF (1977). Land, sea and climate in the northern Adriatic region during Late Pleistocene and Holocene. Palaeogeography, Palaeoclimatology, Palaeoecology.

[R10] Bukowski Z (1988). Critically about the so-called amber route in the Odra and Vistula river basin in the early iron age. Archaeologia Polona.

[R11] Busby JR, Margules CR, Austin MP (1991). BIOCLIM—a bioclimatic analysis and prediction system. Nature conservation: cost effective biological surveys and data analysis.

[R12] Cadahía L, Harl J, Duda M, Sattmann H, Kruckenhauser L, Fehér Z, Zopp L, Haring E (2014). New data on the phylogeny of Ariantinae (Pulmonata, Helicidae) and the systematic position of *Cylindrus obtusus* based on nuclear and mitochondrial DNA marker sequences. Journal of Zoological Systematics and Evolutionary Research.

[R13] Cieślak E (2014). Phylogeography of Pontic-Pannonian species in Central Europe. Polish Botanical Studies.

[R14] Cook LM, Peake JF (1960). A study of some populations of *Cepaea nemoralis* L. from the Dartry Mountains, Co. Sligo, Ireland. Proceedings of the Malacological Society.

[R15] Cserkész T, Rusin M, Sramkó G (2016). An integrative systematic revision of the European southern birch mice (Rodentia: Sminthidae, Sicista subtilis group). Mammal Review.

[R16] Dengler J, Janišová M, Török P, Wellstein C (2014). Biodiversity of Palaearctic grasslands: a synthesis. Agriculture, Ecosystems & Environment.

[R17] Durka W, Nossol C, Ruprecht E, Wagner V, Welk E, Hensen I (2013). Extreme genetic depauperation and differentiation of both populations and species in Eurasian feather grasses (*Stipa*). Plant Systematics and Evolution.

[R18] Egorov R (2014). The first record of *Cepaea vindobonensis* (Pfeiffer, 1828) (Stylommatophora: Helicidae) in the central part of European Russia. Malacologica Bohemoslovaca.

[R19] Ehlers J, Gibbard P (2004). Quaternary glaciations—extent and chronology.

[R20] Elith J, Kearney M, Phillips S (2010). The art of modelling range-shifting species. Methods in Ecology and Evolution.

[R21] Elith J, Phillips SJ, Hastie T, Dudík M, Chee YE, Yates CJ (2011). A statistical explanation of MaxEnt for ecologists. Diversity and Distributions.

[R22] Excoffier L, Lischer HEL (2010). Arlequin suite ver 3.5: a new series of programs to perform population genetics analyses under Linux and Windows. Molecular Ecology Resources.

[R23] Fekete G, Molnár Z, Magyari E, Somodi I, Varga Z (2014). A new framework for understanding Pannonian vegetation patterns: regularities, deviations and uniqueness. Community Ecology.

[R24] Fielding AH, Bell JF (1997). A review of methods for the measurement of prediction errors inconservation presence/absence models. Environmental Conservation.

[R25] Folmer O, Black M, Hoeh W, Lutz R, Vrijenhoek R (1994). DNA primers for amplification of mitochondrial cytochrome c oxidase subunit I from diverse metazoan invertebrates. Molecular Marine Biology and Biotechnology.

[R26] Fourcade Y, Engler JO, Rödder D, Secondi J (2014). Mapping species distributions with MAXENT using a geographically biased sample of presence data: a performance assessment of methods for correcting sampling bias. PloS One.

[R27] Franklin J, Miller J, Franklin J (2009). Statistical methods—modern regression. Mapping species distribution: spatial inference and prediction.

[R28] Galtier N, Nabholz B, Glemin S, Hurst GDD (2009). Mitochondrial DNA as a marker of molecular diversity: a reappraisal. Molecular Ecology.

[R29] Gittenberger E, Piel WH, Groenenberg DSJ (2004). The Pleistocene glaciations and the evolutionary history of the polytypic snail species *Arianta arbustorum* (Gastropoda, Pulmonata, Helicidae). Molecular Phylogenetics and Evolution.

[R30] González-Sampériz P, Leroy S, Carrión J, Fernández S, García-Antón M, Gil-García M, Uzquiano P, Valero-Garcés B, Figueiral I (2010). Steppes, savannahs, forests and hytodiversity reservoirs during the Pleistocene in the Iberian Peninsula. Review of Palaeobotany and Palynology.

[R31] Grindon AJ, Davison A (2013). Irish *Cepaea nemoralis* land snails have a cryptic Franco-Iberian origin that is most easily explained by the movements of Mesolithic humans. PloS One.

[R32] Guindon S, Dufayard JF, Lefort V, Anisimova M, Hordijk W, Gascuel O (2010). New algorithms and methods to estimate maximum-likelihood phylogenies: assessing the performance of PhyML 3.0. Systematic Biology.

[R33] Hadid Y, Németh A, Snir S, Pavlíček T, Csorba G, Major Á, Mezhzherin S, Rusin M, Coşkun Y, Nevo E (2012). Is evolution of blind mole rats determined by climate oscillations?. PloS One.

[R34] Hall TA (1999). BioEdit: a user-friendly biological sequence alignment editor and analysis program for Windows 95/98/NT. Nucleic Acids Symposium Series.

[R35] Harl J, Duda M, Kruckenhauser L, Sattmann H, Haring E (2014). In search of glacial refuges of the land snail *Orcula dolium* (Pulmonata, Orculidae)—an integrative approach using DNA sequence and fossil data. PloS One.

[R36] Hewitt G (2000). The genetic legacy of the quaternary ice ages. Nature.

[R37] Hewitt GM (2004). Genetic consequences of climatic oscillation in the quaternary. Philosophical Transactions of the Royal Society B: Biological Sciences.

[R38] Holderegger R, Thiel-Egenter C (2008). A discussion of different types of glacial refugia used in mountain biogeography and phylogeography. Journal of Biogeography.

[R39] Hurst GDD, Jiggins FM (2005). Problems with mitochondrial DNA as a marker in population, phylogeographic and phylogenetic studies: the effects of inherited symbionts. Proceedings of the Royal Society of London Series B.

[R40] Jesse R, Véla E, Pfenninger M (2011). Phylogeography of a land snail suggests trans-Mediterranean Neolithic transport. PloS One.

[R41] Joger U, Fritz U, Guicking D, Kalyabina-Hauf S, Nagy ZT, Wink M (2007). Phylogeography of western Palaearctic reptiles—spatial and temporal speciation patterns. Zoologischer Anzeiger.

[R42] Kajtoch Ł, Lachowska-Cierlik D, Mazur M (2009). Genetic diversity of xerothermic weevils *Polydrusus inustus* and *Centricnemus leucogrammus* (Coleoptera: Curculionidae) in central Europe. European Journal of Entomology.

[R43] Kajtoch Ł, Cieślak E, Varga Z, Paul W, Mazur MA, Sramkó G, Kubisz D (2016). Phylogeographic patterns of steppe species in eastern Central Europe: a review and the implications for conservation. Biodiversity and Conservation.

[R44] Kerney MP, Cameron RAD, Jungbluth JH (1983). Die Landschnecken Nord- und Mitteleuropas.

[R45] Klemm W (1973). Die Verbreitung der rezenten Land-Gehäuse-Schnecken in Österreich. Denkschriften der Österreichischen Akademie der Wissenschaften.

[R46] Kramarenko SS (2014). Aktivnaya i passivnaya migratsiya nazemnykh mollyuskov: obzor [active and passive dispersal of terrestrial mollusks: a review]. Ruthenica.

[R47] Kryštufek B, Ivanitskaya E, Arslan A, Arslan E, Bužan EV (2012). Evolutionary history of mole rats (genus *Nannospalax*) inferred from mitochondrial cytochrome b sequence. Biological Journal of the Linnean Society.

[R48] Kubisz D, Kajtoch Ł, Mazur MA, Lis A, Holecova M (2012). Conservation genetics of highly isolated populations of xerothermic *Crioceris quatuordecimpunctata* (Coleoptera: Chrysomelidae). Invertebrate Biology.

[R49] Kunica NA (2007). Priroda Ukrainy v plejstocene (po dannym malakofaunisticheskogo analiza) [nature of Ukraine in Pleistocene (by the data of malacofaunistic analysis)].

[R50] Kurtaev MG-K, Magomedova MZ, Kurtaev DM (2012). Distribution of some modern genera and species of terrestrial mollusks in the Tertiary of Daghestan. [Rasprostranenie nekotorykh sovremennykh rodov I vidov nazemnykh mollyusdkov v Dagestane v Tretichnom periode].

[R51] Kwiatkowska K, Manasterski D (2013). Amber routes in Central Europe’s prehistory—an overview.

[R52] Liana A, Baccetti B (1987). Orthoptera of xerothermic habitats in Poland and their origin. Evolutionary biology of Orthopteroid insects.

[R53] Librado P, Rozas J (2009). DnaSP v5: A software for comprehensive analysis of DNA polymorphism data. Bioinformatics.

[R54] Ložek V (1964). Quartärmollusken der Tschechoslovakei. Rozpravy ÚÚG.

[R55] Lubell D (2004). Are land snails a signature for the Mesolithic-Neolithic transition?. Documenta Prehistorica.

[R56] Markova AK, Simakova AN, Puzachenko AY (2009). Ecosystems of Eastern Europe at the time of maximum cooling of the Valdai glaciation (24-18 kyr BP) inferred from data on plant communities and mammal assemblages. Quaternary International.

[R57] Mazur M (2001). Ryjkowce kserotermiczne Polski (Curculionoidea: Nemonychidae, Attelabidae, Apionidae, Curculionidae). Studium zoogeograficzne. Monografie Fauny Polski.

[R58] Mazur M, Kubisz D (2013). Distribution and migration of the xerothermic beetles (Coleoptera) in the Vistula River valley. Monografie Faunistyczne.

[R59] McNeill WH (2011). Europe’s Steppe Frontier, 1500–1800.

[R60] Mierzwa D (2009). *Cepaea vindobonensis* (Férussac, 1821) (Gastropoda: Pulmonata: Helicidae) in central, northwestern and western Poland. Folia Malacologica.

[R61] Mierzwa-Szymkowiak D (2012). Occurrence of *Cepaea vindobonensis* in Poland—new data. Folia Malacologica.

[R62] Nehring A (1890). Ueber Tundren und Steppen der Jetzt-und Vorzeit.

[R63] Neiber MT, Hausdorf B (2015). Molecular phylogeny reveals the polyphyly of the snail genus *Cepaea* (Gastropoda: Helicidae). Molecular Phylogenetics and Evolution.

[R64] Neiber MT, Sagorny C, Hausdorf B (2016). Increasing the number of molecular markers resolves the phylogenetic relationship of ‘*Cepaea*’ *vindobonensis* (Pfeiffer 1828) with Caucasotachea Boettger 1909 (Gastropoda: Pulmonata: Helicidae). Journal of Zoological Systematics and Evolutionary Research.

[R65] Neubert E, Bank RA (2006). Notes on the species of *Caucasotachea* C. Boettger 1909 and *Lindholmia* P. Hesse 1919, with annotations to the Helicidae (Gastropoda: Stylommatophora: Helicidae). Archiv für Molluskenkunde.

[R66] Neumann K, Michaux JR, Maak S, Jansman HAH, Kayser A, Mundt G, Gattermann R (2005). Genetic spatial structure of European common hamsters (Cricetus Cricetus)—a result of repeated range expansion and demographic bottlenecks. Molecular Ecology.

[R67] Paradis E, Claude J, Strimmer K (2004). APE: analyses of phylogenetics and evolution in R language. Bioinformatics.

[R68] Pärtel M, Bruun HH, Sammul M, Lillak R, Viiralt R, Linke A, Geherman V (2005). Biodiversity in temperate European grasslands: origin and conservation. Integrating efficient grass-land farming and biodiversity.

[R69] Pfenninger M, Posada D (2002). Phylogeographic history of the land snail *Candidula unifasciata* (Helicellinae, Stylommatophora): fragmentation, corridor migration, and secondary contact. Evolution.

[R70] Pfenninger M, Posada D, Magnin F (2003). Evidence for survival of Pleistocene climatic changes in northern refugia by the land snail *Trochoidea geyeri* (Soos 1926) (Helicellinae, Stylommatophora). Biomedcentral Evolutionary Biology.

[R71] Phillips SJ, Anderson RP, Schapire RE (2006). Maximum entropy modeling of species geographic distributions. Ecological Modelling.

[R72] Pokorný P, Chytrý M, Juřičková L, Sádlo J, Novák J, Ložek V (2015). Mid-Holocene bottleneck for central European dry grasslands: did steppe survive the forest optimum in northern bohemia, Czech Republic?. The Holocene.

[R73] Przybyłowicz Ł, Lukhtanov V, Lachowska-Cierlik D (2014). Towards the understanding of the origin of the Polish remote population of *Polyommatus* (*Agrodiaetus*) *ripartii* (Lepidoptera: Lycaenidae) based on karyology and molecular phylogeny. Journal of Zoological Systematics and Evolutionary Research.

[R74] R Development Core Team (2014). R: a language and environment for statistical computing.

[R75] Ree R, Smith S (2008). Maximum likelihood inference of geographic range evolution by dispersal, local extinction, and cladogenesis. Systematic Biology.

[R76] Ribera I, Blasco-Zumeta J (1998). Biogeographical links between steppe insects in the Monegros region (Aragon, NE Spain), the eastern Mediterranean, and central Asia. Journal of Biogeography.

[R77] Richards PM, Liu MM, Lowe N, Davey JW, Blaxter ML, Davison A (2013). RAD-Seq derived markers flank the shell colour and banding loci of the *Cepaea nemoralis* supergene. Molecular Ecology.

[R78] Rogers AR, Harpending H (1992). Population growth makes waves in the distribution of pairwise genetic differences. Molecular Biology and Evolution.

[R79] Rutkowski R, Sielezniew M, Szostak A (2009). Contrasting levels of polymorphism in cross-amplified microsatellites in two endangered xerothermophilous, obligatorily myrmecophilous, butterflies of the genus *Phengaris* (Maculinea) (Lepidoptera: Lycaenidae). European Journal of Entomology.

[R80] Schikov EV (2016). Adventivnye vidy nazemnoj malakofauny centra Russkoj ravniny [adventive species of terrestrial malacofauna in the central portion of the Russian plain]. Ruthenica.

[R81] Schileyko AA, Rymzhanov TS (2013). Fauna of land mollusks (Gastropoda, Pulmonata Terrestria) of Kazakhstan and adjacent territories.

[R82] Schmitt T (2007). Molecular biogeography of Europe: Pleistocene cycles and postglacial trends. Frontiers in Zoology.

[R83] Schmitt T (2009). Biogeographical and evolutionary importance of the European high mountain systems. Frontiers in Zoology.

[R84] Schmitt T, Varga Z (2012). Extra-Mediterranean refugia: the rule and not the exception?. Frontiers in Zoology.

[R85] Schönswetter P, Stehlik I, Holderegger R, Tribsch A (2005). Molecular evidence for glacial refugia of mountain plants in the European Alps. Molecular Ecology.

[R86] Scoble J, Lowe AJ (2010). A case for incorporating phylogeography and landscape genetics into species distribution modelling approaches to improve climate adaptation and conservation planning. Diversity and Distributions.

[R87] Snegin EA, Snegina EA (2016). The genetic structure of populations of specially protected mollusk *Cepaea vindobonensis* (Mollusca, Gastropoda, Pulmonata) in a north-eastern part of the modern area. Ecological Genetics.

[R88] Soós L (1943). A Kárpát–medence Mollusca faunája. [the Mollusc Fauna of the Carpathian Basin].

[R89] Stewart JR, Lister AM (2001). Cryptic northern refugia and the origins of the modern biota. Trends in Ecology & Evolution.

[R90] Stewart JR, Lister AM, Barnes I, Dalén L (2010). Refugia revisited: Individualistic responses of species in space and time. Proceedings of the Royal Society B, Biological Sciences.

[R91] Stohlgren TJ, Ma P, Kumar S, Rocca M, Morisette JT, Jarnevich CS, Benson N (2010). Ensemble habitat mapping of invasive plant species. Risk Analysis.

[R92] Swets JA (1988). Measuring the accuracy of diagnostic systems. Science.

[R93] Szczecińska M, Sramko G, Wołosz K, Sawicki J (2016). Genetic diversity and population structure of the rare and endangered plant species *Pulsatilla patens* (L.) Mill in East Central Europe. PloS One.

[R94] Taberlet P, Fumagalli L, Wust-Saucy AG, Cossons JF (1998). Comparative phylogeography and postglacial colonization routes in Europe. Molecular Ecology.

[R95] Tamura K, Stecher G, Peterson D, Filipski A, Kumar S (2013). MEGA6: Molecular Evolutionary Genetics Analysis version 6.0. Molecular Biology and Evolution.

[R96] Thompson JD, Gibson TJ, Plewniak F, Jeanmougin F, Higgins DG (1997). The ClustalX windows interface: flexible strategies for multiple sequence alignment aided by quality analysis tools. Nucleic Acids Research.

[R97] Toews DPL, Brelsford A (2012). The biogeography of mitochondrial and nuclear discordance in animals. Molecular Ecology.

[R98] Varga Z, Habel JC, Assmann T (2010). Extra-Mediterranean refugia, post-glacial vegetation history and area dynamics in eastern Central Europe. Relict species, phylogeography and conservation biology.

[R99] Varga ZS, Schmitt T (2008). Types of oreal and oreotundral disjunction in the western Palearctic. Biological Journal of the Linnean Society.

[R100] Wahlberg N, Saccheri I (2007). The effects of Pleistocene glaciations on the phylogeography of *Melitaea cinxia* (Lepidoptera: Nymphalidae). European Journal of Entomology.

[R101] Warren DL, Seifert SN (2010). Ecological niche modeling in Maxent: the importance of model complexity and the performance of model selection criteria. Ecological Applications.

[R102] Warren DL, Glor RE, Turelli M (2010). ENMTools: a toolbox for comparative studies of environmental niche models. Ecography.

[R103] Welter-Schultes FW (2008). Bronze age shipwreck snails from Turkey: first direct evidence for oversea carriage of land snails in antiquity. Journal of Molluscan Studies.

[R104] Welter-Schultes FW (2012). European non-marine molluscs, a guide for species identification.

[R105] Willis KJ, van Andel TA (2004). Trees or no trees? The environments of central and eastern Europe during the last glaciation. Quaternary Science Reviews.

[R106] Yurtsev BA, Hopkins DM, Matthews JV, Schweger CE (1982). Relics of the xerophyte vegetation of Beringia in northeastern Asia. Palaeoecology of Beringia.

[R107] Yurtsev BA (2000). The Pleistocene “tundra-steppe” and the productivity paradox: the landscape approach. Quaternary Science Reviews.

