# Supplementary figures and images for: Reconstructed historical distribution and phylogeography unravels non-steppic origin of Caucasotachea vindobonensis (Gastropoda: Helicidae)
Source: Org Divers Evol. Author manuscript; Available in PMC 2018 May 23. (PMC5965669; doi:10.1007/s13127-017-0337-3)

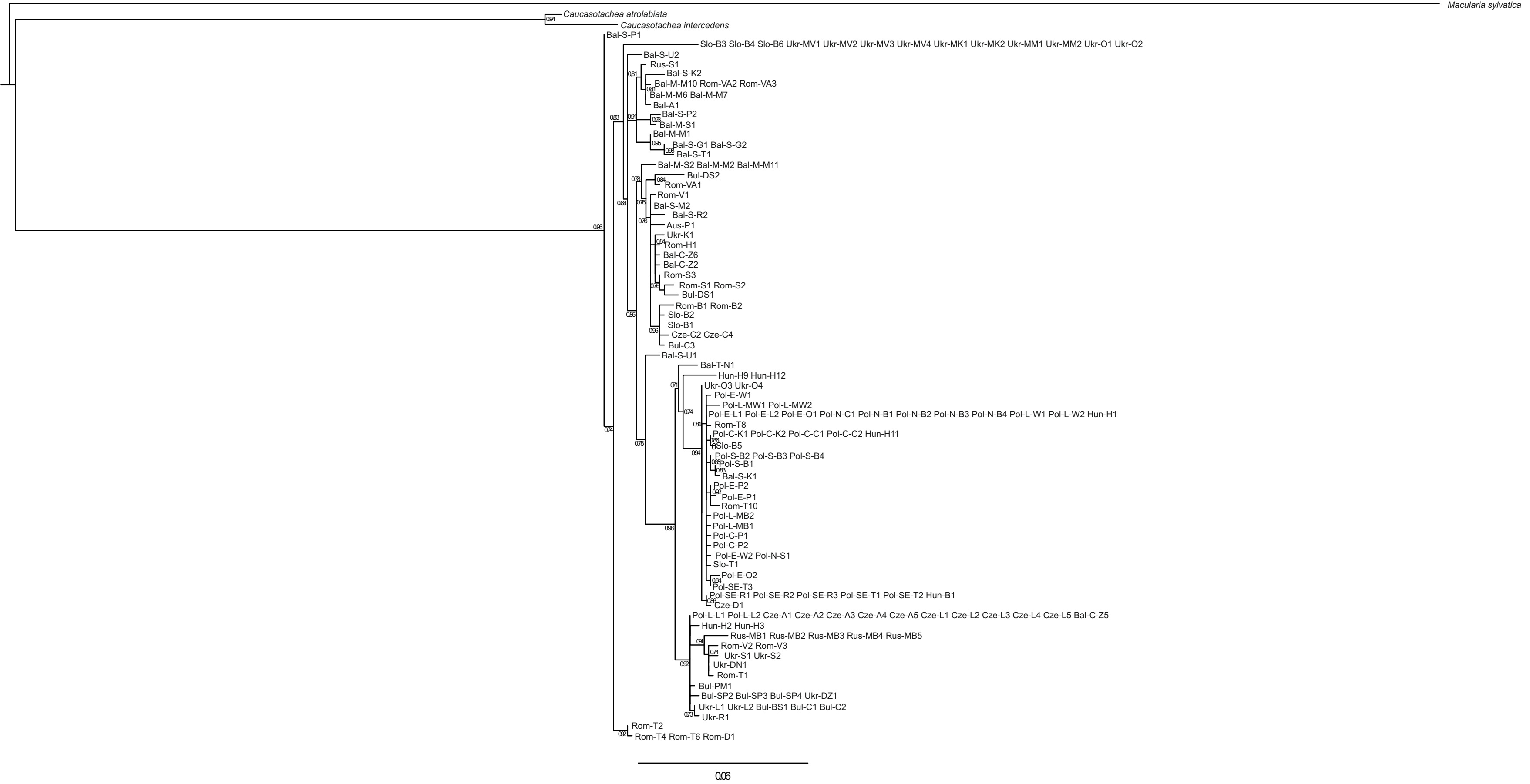

Supplement: Supplementary figure 1 — Maximum likelihood phylogenetic tree reconstructed on haplotypes found in Caucasotachea vindobonensis populations. Values indicate branch supports [file NIHMS77630-supplement-Supplementary_figure_1.pdf]

BALKAN

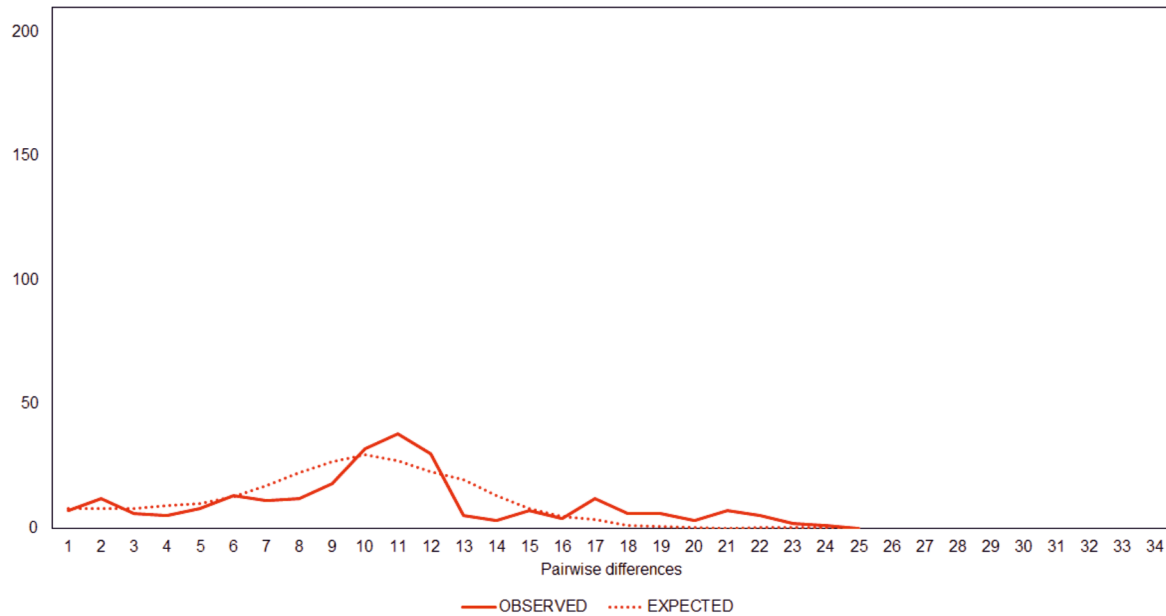

CARPATHO-PANNONIAN

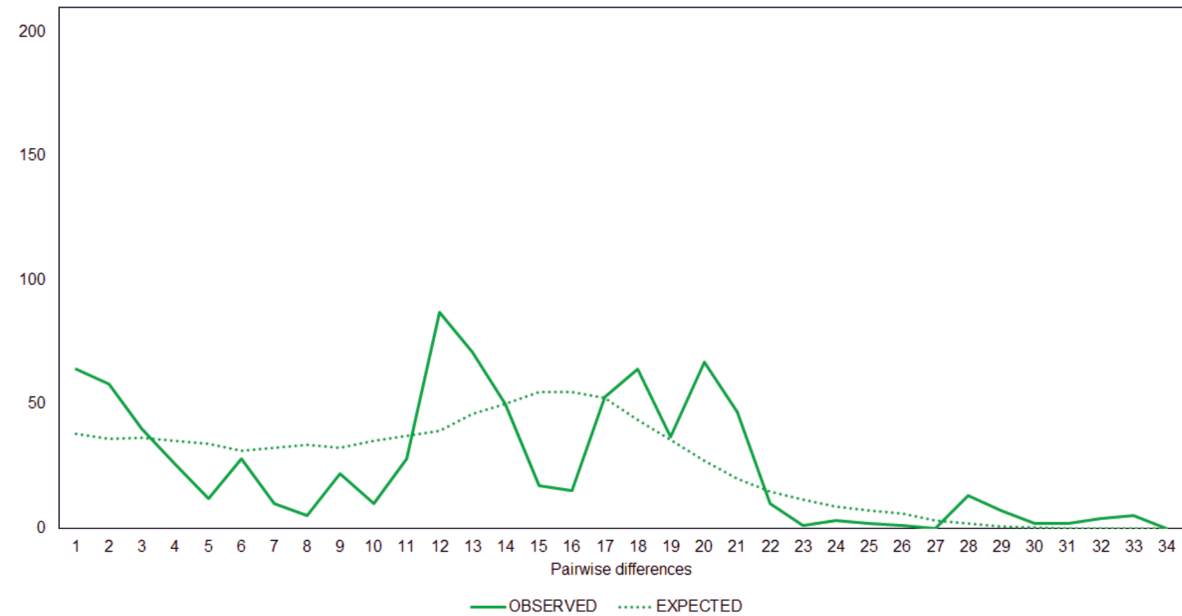

NORTH-WESTERN

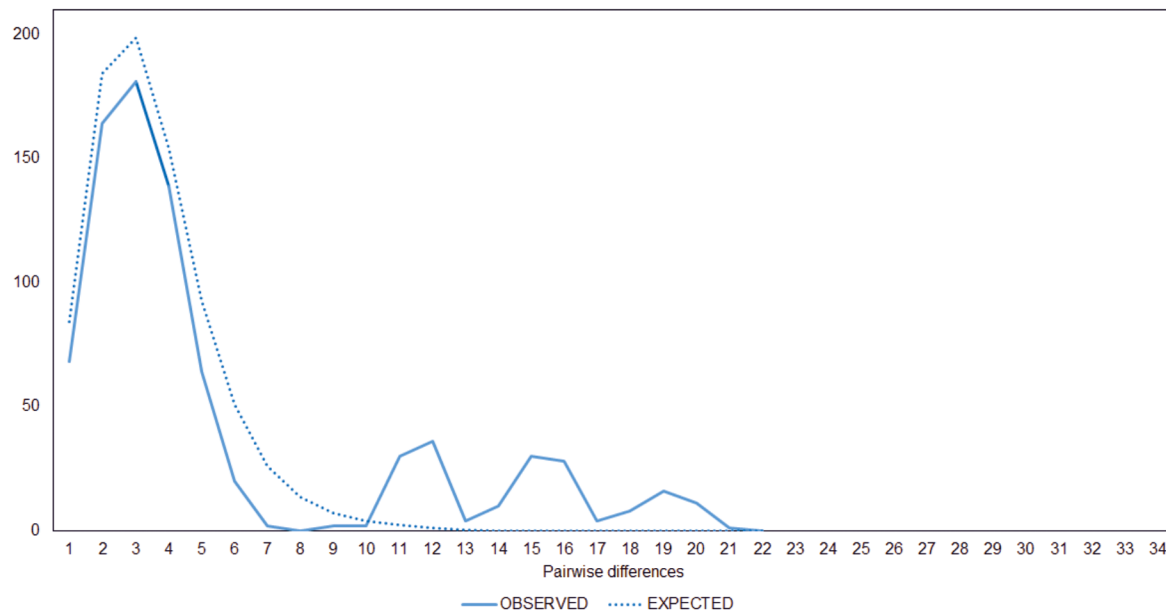

PONTO-CASPIAN

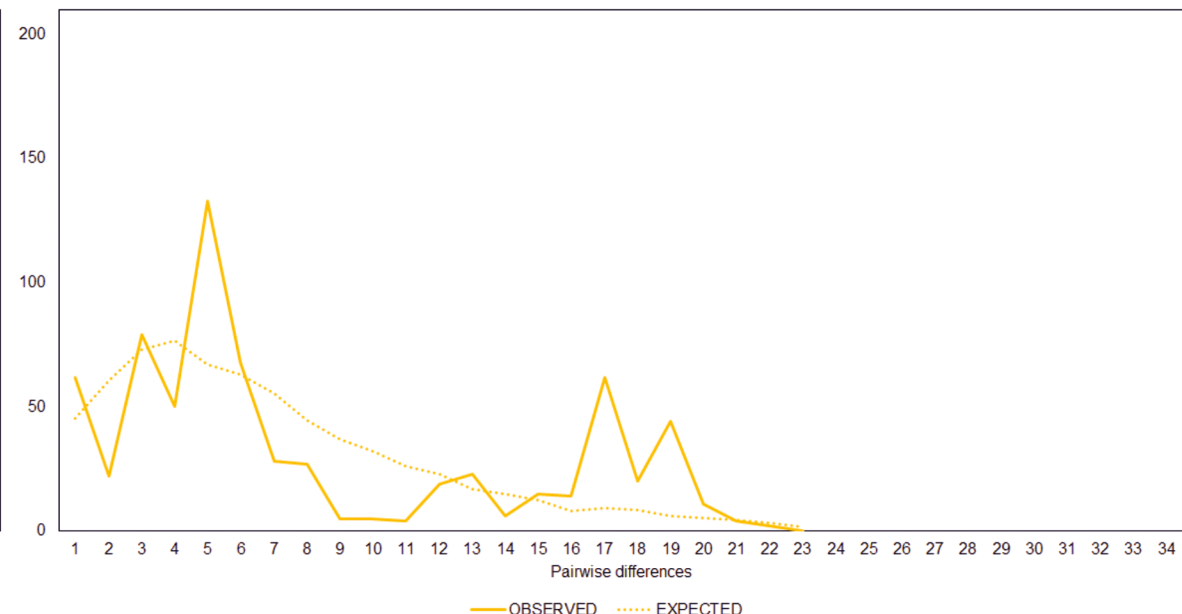

Supplement: Supplementary figure 2 — Mismatch distribution calculated for four regional groups of Caucasotachea vindobonensis populations [file NIHMS77630-supplement-Supplementary_figure_2.pdf]

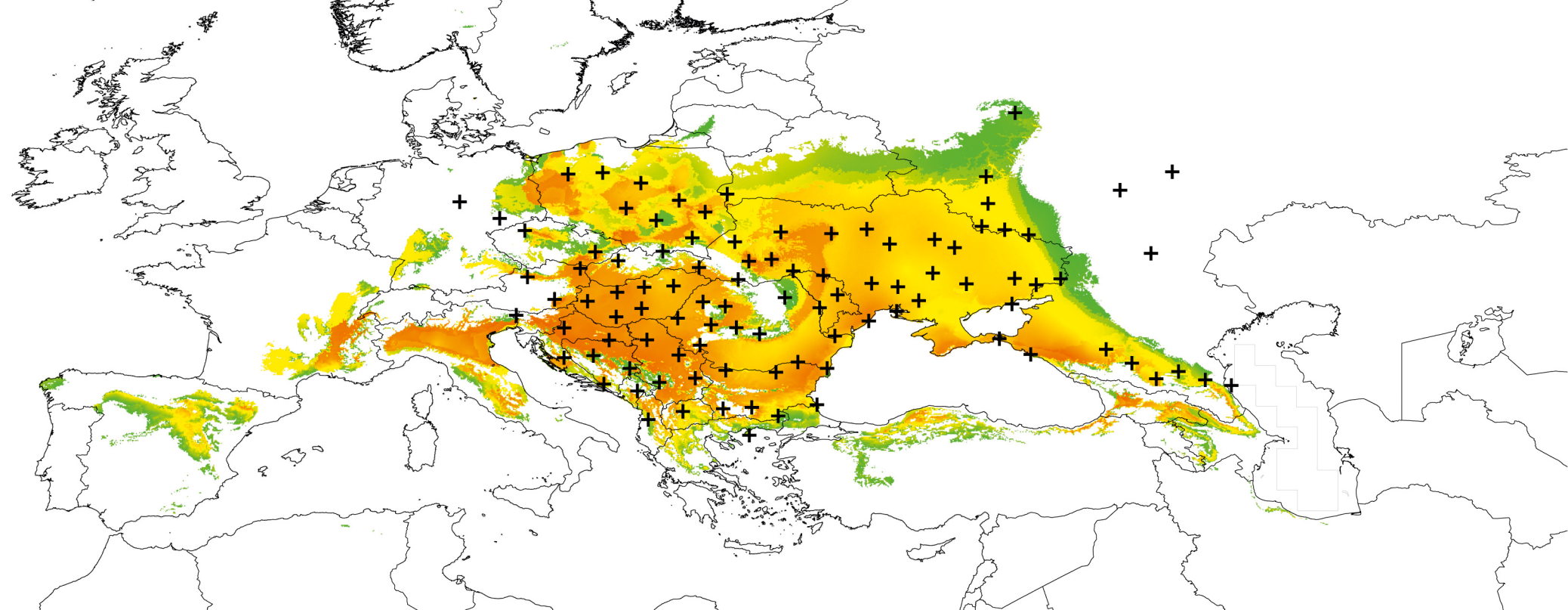

Supplement: Supplementary figure 3 — The potential distribution of Caucasotachea vindobonensis. Warmer colors indicate more suitable climatic conditions, while crosses indicate the subsample of presence points used for modeling (for more detail see material and methods) [file NIHMS77630-supplement-Supplementary_figure_3.pdf]
