# Supplementary material for: Reconstructed historical distribution and phylogeography unravels non-steppic origin of Caucasotachea vindobonensis (Gastropoda: Helicidae)
Source: Org Divers Evol. Author manuscript; Available in PMC 2018 May 23. (PMC5965669; doi:10.1007/s13127-017-0337-3)
Supplement: Supplementary table 4 [file NIHMS77630-supplement-Supplementary_table_4.docx]

Supplementary Table 4. Analysis of the pairwise differences distribution (Mismatch distribution) calculated with use of COI sequences for four regional groups of *Caucasotachea vindobonensis* populations.

|  | Balkan | Carpatho- | Ponto- | North- |
| --- | --- | --- | --- | --- |
|  |  | Pannonian | Caspian | Western |
| demographic expansion | | | | |
| Tau | 16.55 | 10.22 | 2.20 | 1.46 |
| Tau qt 5% | 5.47 | 5.81 | 0.22 | 0.61 |
| Tau qt 95% | 22.87 | 11.98 | 16.77 | 4.02 |
| Theta0 | 0.04 | 0.00 | 5.32 | 1.06 |
| Theta0 qt 5% | 0.00 | 0.00 | 0.00 | 0.00 |
| Theta0 qt 95% | 2.83 | 1.84 | 6.06 | 1.47 |
| Theta1 | 21.41 | 31.67 | 17.15 | 99999.00 |
| Theta1 qt 5% | 13.39 | 16.90 | 10.64 | 10.66 |
| Theta1 qt 95% | 65.88 | 501.36 | 99999.00 | 99999.00 |
| SSD | 0.02 | 0.01 | 0.03 | 0.01 |
| Model (SSD) p-value | 0.32 | 0.26 | 0.28 | 0.17 |
| Raggedness index | 0.02 | 0.02 | 0.05 | 0.03 |
| Raggedness p-value | 0.29 | 0.52 | 0.11 | 0.60 |
| spatial expansion | | | | |
| Tau | 10.55 | 7.42 | 2.00 | 1.45 |
| Tau qt 5% | 5.66 | 4.96 | 0.96 | 0.85 |
| Tau qt 95% | 25.57 | 12.00 | 25.10 | 3.13 |
| Theta | 6.27 | 3.31 | 7.06 | 1.08 |
| Theta qt 5% | 0.09 | 0.00 | 0.00 | 0.00 |
| Theta qt 95% | 12.52 | 9.21 | 10.84 | 2.89 |
| M | 3.24 | 18.86 | 7.23 | 99999.00 |
| M qt 5% | 1.34 | 7.29 | 0.68 | 9.76 |
| M qt 95% | 14.50 | 69.10 | 441.89 | 99999.00 |
| SSD | 0.01 | 0.02 | 0.02 | 0.01 |
| Model (SSD) p-value | 0.74 | 0.38 | 0.51 | 0.15 |
| Raggedness index | 0.02 | 0.02 | 0.02 | 0.03 |
| Raggedness p-value | 0.80 | 0.71 | 0.66 | 0.65 |
