# Supplementary material for: Reconstructed historical distribution and phylogeography unravels non-steppic origin of Caucasotachea vindobonensis (Gastropoda: Helicidae)
Source: Org Divers Evol. Author manuscript; Available in PMC 2018 May 23. (PMC5965669; doi:10.1007/s13127-017-0337-3)
Supplement: Supplementary table 5 [file NIHMS77630-supplement-Supplementary_table_5.docx]

Supplementary Table 5. Results of model selection based on Akaike Information Criterium. The numbers in the model names indicate BIOCLIM variables (Busby 1991) while alt is abbreviation of altitude.

| Model | Log Likelihood | Parameters | delta AIC | Sample Size | AIC score | AICc score | BIC score |
| --- | --- | --- | --- | --- | --- | --- | --- |
| 7-10-15-18 | -1250.3 | 28.0 | 0.0 | 100.0 | 2556.6 | 2579.5 | 2629.6 |
| 7-10-12-15-17-19 | -1245.7 | 35.0 | 4.7 | 100.0 | 2561.3 | 2600.7 | 2652.5 |
| 1-7-12-15-17-19 | -1246.3 | 36.0 | 8.0 | 100.0 | 2564.6 | 2606.9 | 2658.4 |
| 7-10-15-17-18-19 | -1245.5 | 37.0 | 8.4 | 100.0 | 2565.1 | 2610.4 | 2661.4 |
| alt-1-12-17 | -1268.2 | 38.0 | 55.7 | 100.0 | 2612.4 | 2661.0 | 2711.4 |
